# Comparison of curative effect between OBS assisted by 3D printing and PFNA in the treatment of AO/OTA type 31-A3 femoral intertrochanteric fractures in elderly patients

**DOI:** 10.3389/fmed.2023.1234764

**Published:** 2023-08-04

**Authors:** Jiazheng Huang, Ying Xiong, Md Miftahul Mithu, Jinping Li, Chengkui Geng, Jipeng Lu, Yunfeng Ren, Ze Yang, Xuewen Gan, Aili Zhang, Huiqin Yang, Zhuoyuan Chen

**Affiliations:** ^1^Key Laboratory of Tumor Immunological Prevention and Treatment of Yunnan Province, Yan’an Hospital Affiliated to Kunming Medical University, Kunming, Yunnan, China; ^2^Department of Orthopedics of Yan’an Hospital Affiliated to Kunming Medical University, Kunming, Yunnan, China; ^3^The Affiliated Changsha Central Hospital, Hengyang Medical School, University of South China, Changsha, Hunan, China

**Keywords:** femoral intertrochanteric fracture, Ortho-Bridge System (OBS), proximal femoral nail anti-rotation (PFNA), 3D printing, guide plate

## Abstract

**Objective:**

To compare and analyze the Ortho-Bridge System (OBS) clinical efficacy assisted by 3D printing and proximal femoral nail anti-rotation (PFNA) of AO/OTA type 31-A3 femoral intertrochanteric fractures in elderly patients.

**Methods:**

A retrospective analysis of 25 elderly patients diagnosed with AO/OTA type 31-A3 femoral intertrochanteric fracture was conducted from January 2020 to August 2022 at Yan’an Hospital, affiliated to Kunming Medical University. The patients were divided into 10 patients in the OBS group and 15 in the PFNA group according to different surgical methods. The OBS group reconstructed the bone models and designed the guide plate by computer before the operation, imported the data of the guide plate and bone models into a stereolithography apparatus (SLA) 3D printer, and printed them using photosensitive resin, thus obtaining the physical object, then simulating the operation and finally applying the guide plate to assist OBS to complete the operation; the PFNA group was treated by proximal femoral nail anti-rotation. The operation time, the intraoperative blood loss, Harris hip score (HHS), Oxford Hip Score (OHS), and complications were compared between the two groups.

**Results:**

The operation time and the intraoperative blood loss in the PFNA group were less than that in the OBS group, and there was a significant difference between the two groups (*P* < 0.05). The HHS during the 6th month using OBS was statistically higher than PFNA (*P* < 0.05), however, there were no significant differences in OHS during the 6th month between the OBS group and PFNA group (*P* > 0.05). The HHS and OHS during the 12th month in the OBS group were statistically better than in the PFNA group (*P* < 0.05).

**Conclusion:**

The OBS assisted by 3D printing and PFNA are effective measures for treating intertrochanteric fractures. Prior to making any decisions regarding internal fixation, it is crucial to evaluate the distinct circumstances of each patient thoroughly.

## 1. Introduction

An intertrochanteric fracture of the femur occurs above the lesser trochanter at the base of the femoral neck (1). According to numerous studies, various complications from fractures can increase the risk of death in elderly patients (2).

Many orthopedic doctors have recently recognized the significance of the lateral wall of the femoral trochanter, and it is believed to be another vital structure that affects the stability of intertrochanteric fractures. The integrity of this structure plays a crucial role in preventing fixation failure and reoperation of intertrochanteric fractures (3–5).

**FIGURE 1:**
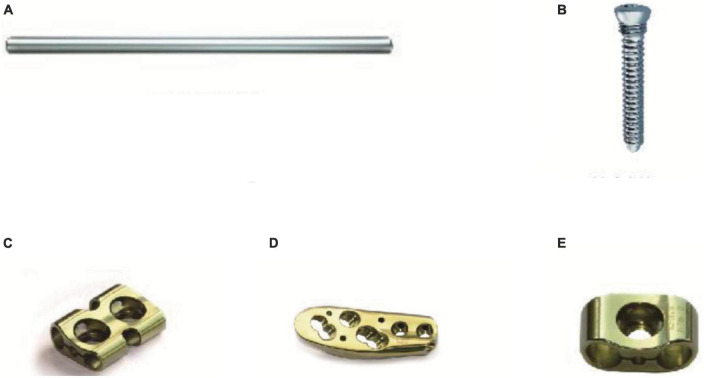
The composition of OBS. **(A)**: The rod of OBS; **(B)**: The screw; **(C)**, and **(E)**: The mass of OBS; **(D)**: The proximal femur anatomic mass of OBS.

Ortho-Bridge System (OBS) is a combined internal fixation system that consists of various sizes of rods, masses, and screws, which can be applied in a myriad of variations such as single-rod, double-rod, and mixed-rod systems (Figure 1). This method combines the advantages of traditional bridging plates, intramedullary nails, and external fixation stents, which have been successfully applied in the fixation of extremity fractures and pelvic fractures (6, 7).

With the constant evolution of 3D printing technology, the applications for personalized surgical guides and bone models are also expanding. Due to the versatility and malleability of OBS, the configuration of nail placement channels and the shaping of internal fixation instruments can be built flawlessly before surgery, which ensures a successful and precise implant placement and, thus, a safe and stable fixation. With all its conveniences, this system can bring more amenities and advantages to orthopedic surgeons, posing a promising future in developing treatment modalities for fractures.

This analysis compares and analyzes the clinical therapeutic effects of OBS assisted by 3D printing and PFNA of AO/OTA type 31-A3 femoral intertrochanteric fractures in elderly patients and provides a theoretical basis for clinical and surgical treatment. The study stands as follows.

## 2. Materials and methods

### 2.1. Research objects

Patients with Fractures admitted to Yan’an Hospital Affiliated to Kunming Medical University from January 2020 to August 2022 were chosen as the study object. Patient data were collected with consent, and patient selection criteria were as follows: age >60 years; AO/OTA type 31-A3; no other systemic disease; all patients could walk unaided or with crutches before fracture. Patients with pathological fractures and surgical contraindications were excluded from this study. The patients were separated into the OBS and PFNA groups according to treatment methods, with 10 and 15 cases in each group, respectively. All selected patients were diagnosed with an intertrochanteric fracture, confirmed by X-ray.

Among 25 patients, 12 were males, and 13 were females, ages ranging from 60 to 87 years, with an average of 70.04 years of age. In total, 13 and 12 cases were left and right inter-trochanteric fractures, respectively. The number of patients with a unique history of injury was as follows, Automobile casualties - 3, Fall from height - 1, and Crash injury - 21.

There was no considerable difference in the general data of the two groups of patients (*P* > 0.05), and they were comparable (Table 1).

**TABLE 1:** Patient demographics.

Factor	OBS group (*n* = 10)	PFNA group (*n* = 15)	*P*-value
**Gender**			
Male	4 (40.00)	8 (53.33)	0.688
Female	6 (60.00)	7 (46.67)	
Age (mean ± SD, y)	69.80 ± 10.10	70.20 ± 7.82	0.912
**Side of fracture**			
Left	4 (40.00)	9 (60.00)	0.284
Right	6 (60.00)	6 (40.00)	
**Cause of injury**			
Crash injury	8 (80.00)	13 (86.67)	0.730
Automobile casualties	1 (10.00)	2 (13.33)	
Fall from height	1 (10.00)	0 (0.00)	

### 2.2. Preparation of 3D printed guide plate and bone model

**FIGURE 2:**
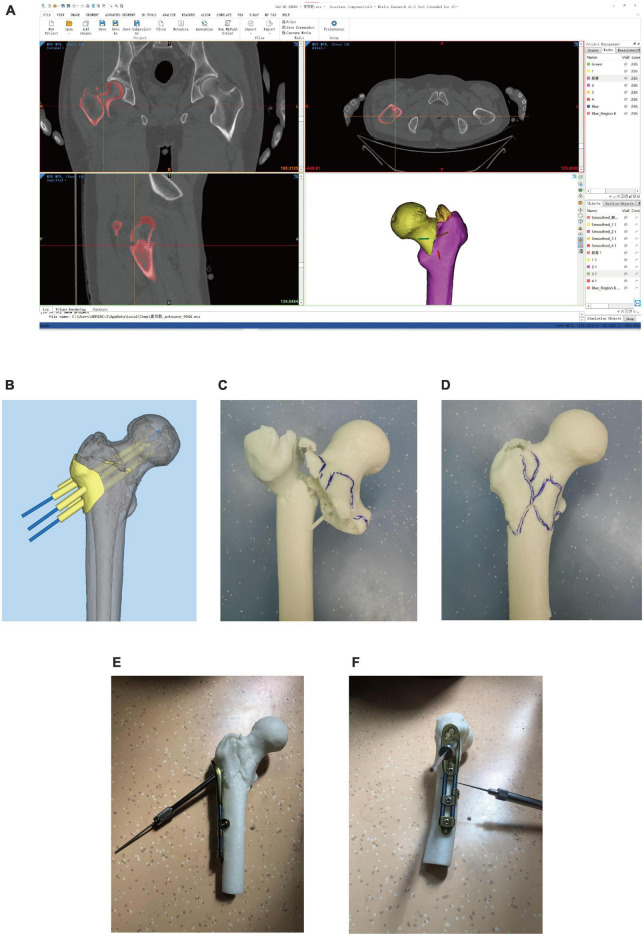
Surgical simulation and the Preparation of 3D printed models. **(A)**: Reconstructed the bone models; **(B)**: Designed the guide plate; **(C,D)**: Bone models; **(E,F)**: Simulated operation.

Pre-operative CT scans of each patient were collected and exported in DICOM format and then imported into Mimics Ver-21.0 software (Materialize, Belgium). This software was used for bone model reconstruction and surface repairs, and the data were exported in STL format. The STL model data of OBS and bone were imported into Magics Ver-21.0 software (Materialize, Belgium) to simulate the placement of the screws in the neck of the femur. 3-Matic Ver-13.0 Software (Materialize, Belgium) was utilized to assemble a guide plate surface, and the Boolean operations were performed to construct the complete guide plate. The data imported into the 3D printer (UnionTech Lite450HD) were used to print the guide plate and bone model (Figure 2).

### 2.3. Surgical methods

**FIGURE 3:**
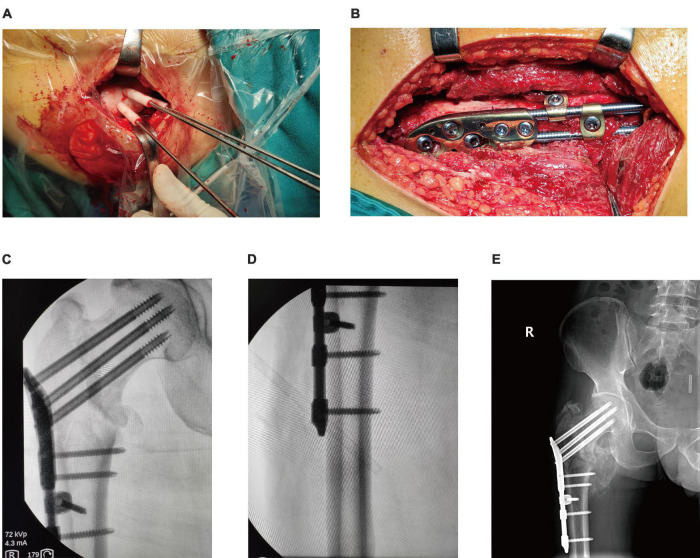
Operation process of OBS assisted by 3D printing. **(A)**: Inserted Kirschner wire through the guide plate to confirm the location of the screws; **(B)**: Observed internal fixation; **(C,D)**: Intraoperative fluoroscope to observe the effect of internal fixation; **(E)**. Postoperative fluoroscopy.

➀ Surgical methods of the OBS group: The OBS group patients underwent surgical methods that involved combined spinal-epidural anesthesia and then positioned supine. The medical procedure involved using a C-arm X-ray machine to perform a closed reduction. The procedure entailed making a longitudinal incision about 6 cm below the greater trochanter to expose the vastus lateralis ridge. A guide plate was placed in the designated position, and three Kirschner wires were drilled along the path to fix the guide plate (Figure 3A). Fluoroscopy was done to ensure that the Kirschner wires were located within the femoral neck and parallel to the middle axis of the femoral neck. After removing the guide plate, the proximal femur anatomic mass was placed along the Kirschner wires, and holes were drilled for screws. The position of the screws and OBS was determined by fluoroscopy (Figures 3C, D), and the incision was washed with physiological saline before suturing (Figure 3B).

➁ Surgical methods of the PFNA group: The patients were given a combination of spinal-epidural anesthesia and positioned on supine position. The closed reduction procedure was aided by the C-arm X-ray machine. A 5 cm incision was made at the top of the greater trochanter, and a guide nail was inserted into the femoral medullary cavity. After expansion of the medullary, an appropriate PFNA main nail was placed and confirmed using fluoroscopy. A guide nail was then inserted into the femoral neck, and a spiral blade was inserted following its direction once the positioning was correct. The distal nail was locked with guidance, and finally, the tail cap of the leading nail was installed. The patient’s incision was washed with physiological saline and sutured to complete the procedure.

➂ Antibiotics and low molecular weight heparin sodium were administered to the patient after the operation. The medical staff guided the patients in rehabilitation exercises. Patients were followed up by outpatient review and telephone.

### 2.4. OHS (Oxford Hip Score)

The OHS is an evaluation method created in 1996 that focuses on the patient’s perspective in assessing pain and functional ability (8, 9). It consists of 12 questions, rated on a scale of 1 to 5, with 1 representing the best outcome and 5 the worst. The sum of the scores from the 12 questions gives a total score ranging from 12 to 60, where 12 represents the best and 60 the worst outcome.

### 2.5. HHS (Harris hip score)

The HHS was initially developed in 1969 to evaluate how the hip joint function is restored in patients after surgery (10). This comprehensive assessment encompasses four key aspects: pain, function, deformity, and joint range of motion. The score is 100, with 90 or more being excellent, 80–89 being good, 70–79 being fair, and less than 70 being poor.

### 2.6. Statistical methods and observation items

For analysis purposes, the statistical software SPSS 26.0 was utilized. The data for measurements (age, operation time, intraoperative blood loss, OHS, HHS) were presented as “x ± s.” We conducted a comparison between groups using either a two-sample *t*-test or Mann–Whitney U-test. For enumeration data (gender, side of fracture, causes of injury) were presented as “%.” Comparison between groups was made using Fisher’s exact test, and a *P*-value of less than 0.05 was considered statistically significant. Additionally, we observed and recorded any complications that arose.

## 3. Results

### 3.1. Comparison of observation indicators between the two groups

**FIGURE 4:**
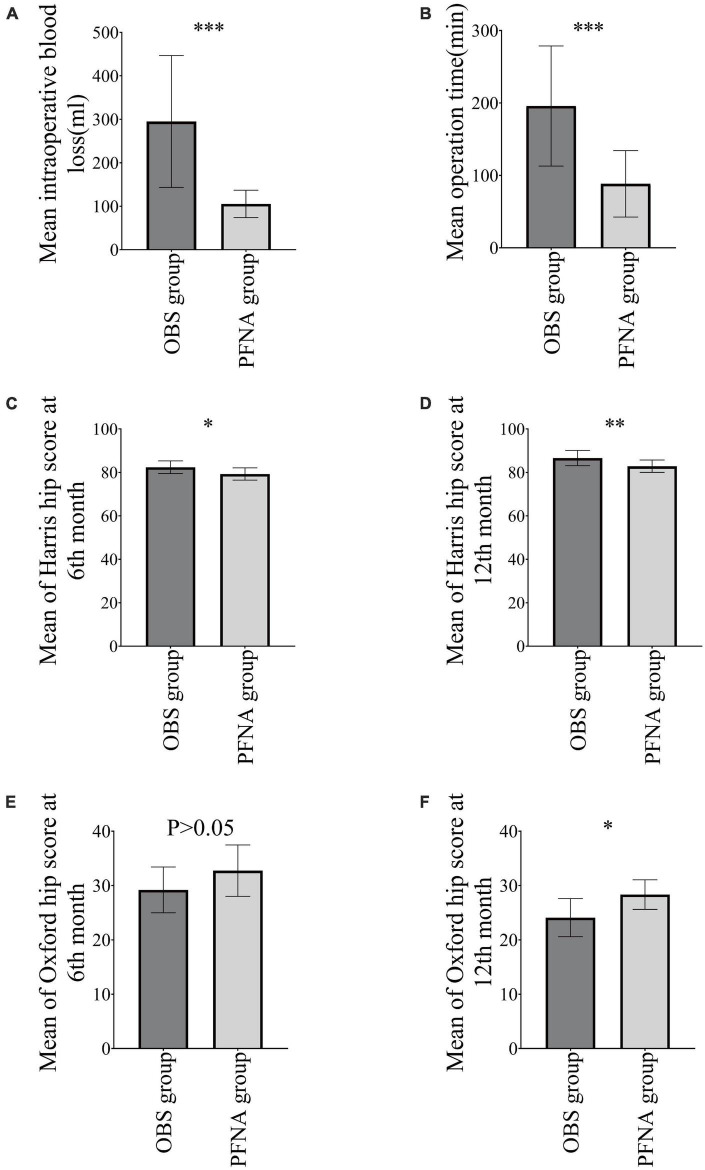
Postoperative Indicators. **(A)** Operation time; **(B)** intraoperative blood loss; **(C)**: HHS at 6th month; **(D)**: OHS at 6th month; **(E)**: HHS at 12th month; **(F)**: OHS at 12th month. *P < 0.05, **P < 0.01, and ***P < 0.001.

The mean operative time and intraoperative blood loss using OBS, as shown in, were 195.80 ± 82.89 min and 295.00 ± 151.75 ml, respectively, which were statistically higher than those in the PFNA group (88.40 ± 45.81 min and 105.33 ± 31.59 ml, *P* < 0.05) (Figures 4A, B).

### 3.2. Comparison of postoperative indicators between the two groups

The HHS in the 6th month using OBS (82.40 ± 2.91) was statistically higher than PFNA (79.27 ± 2.82) (*P* < 0.05), but there was no significant difference in OHS in the 6th month between the OBS group (29.20 ± 4.21) and PFNA group (27.84 ± 5.03) (*P* > 0.05). The HHS and OHS in the 12th month using OBS were 86.60 ± 3.50 and 24.10 ± 3.51, respectively, which were statistically better than those in the PFNA group (82.87 ± 2.85 and 28.33 ± 2.72, *P* < 0.05) (Figures 4C–F).

### 3.3. Comparison of postoperative complications between the two groups

The potential complications of the procedure include infection, deep vein thrombosis, implant migration, and reoperation (Table 2). A total of 1 case of wound infection was reported in the PFNA group, but it was resolved with anti-infection medication and wound care. The OBS and PFNA groups had 1 and 2 cases of deep vein thrombosis, respectively.

**TABLE 2:** Postoperative complications *n* (%).

Factor	OBS group (*n* = 10)	PFNA group (*n* = 15)
Total	1 (10.00)	2 (13.33)
Infection	0	1 (6.67)
Deep venous thrombosis	1 (10.00)	1 (6.67)
Implant migration	0	0
Reoperation	0	0

## 4. Discussion

According to clinical observation, intertrochanteric fractures account for about 3–4% of total body fractures, 24.56% of femur fractures, and 50% of proximal femur fractures, and its treatment method is mainly surgical treatment (1). Femoral intertrochanteric fractures are more common in elderly patients (2, 11). Surgical treatment mainly includes internal fixation (intramedullary fixation, extramedullary fixation), external fixation brace, artificial hip replacement, Sheehan et al. (12). Common intramedullary fixation includes PFN, PFNA, Gamma, and InterTAN, and extramedullary fixation includes DHS, DCS, and PCCP (12–15). In recent years, many experts have conducted clinical studies on intramedullary and extramedullary fixation. However, they have been inconclusive, and all the various surgical approaches have certain advantages in different aspects (16).

Most patients with hip fractures are elderly, and the medial side is less likely to provide adequate support, and the fracture instability may increase in the absence of the lateral wall, thus potentially affecting surgical outcomes (3, 17). However, the treatment modality for intertrochanteric fractures of the femur remains controversial; regardless of the modality chosen, the lateral wall should be protected to the greatest extent possible to avoid lateral wall injury, thus contributing to a higher rate of surgery success (18).

Presently, PFNA is widely used in treating intertrochanteric fractures of the femur (19). Although satisfactory clinical outcomes have been reported, internal fixation failure still occurs in some cases, adversely affecting fracture healing, especially for unstable intertrochanteric fractures (20, 21). The complications associated with PFNA include screw cut-outs and cut-ins, any blade migration irrespective of the direction, telescoping effect, loss of reduction, and implant fracture, et al., which are critical and should not be overlooked (19). In order to avoid these complications, meticulous preoperative planning, intraoperative efforts to achieve anatomic repositioning as thoroughly as possible, selection of appropriate intramedullary nails, and precise control of the tip-apex distance are essential (22, 23). Some clinical workers have proposed that when the fracture involves a lateral wall comminuted fracture, using intramedullary nailing combined with a plate can enhance the stability of the lateral wall and attain favorable outcomes (24).

As a new orthopedic internal fixation device, the modular design of OBS allows flexible fixation and effectively avoids stress masking; the multi-directional placement of screws achieves a three-dimensional fixation effect and exerts strong anti-shear, anti-rotation, and anti-bending impact, with good biomechanical properties (25, 26). OBS can provide better fixation by adding fixation masses and rods for comminuted fractures of the greater trochanter and lateral wall fractures. Through experimental studies, it was found that OBS can significantly reduce the damage to the blood supply at the fracture end and promote fracture healing while ensuring fracture fixation and avoiding stress masking (27). Some clinicians have achieved satisfactory clinical outcomes by utilizing OBS as a treatment modality for intertrochanteric fractures of the femur (28, 29).

As digital technology continues to evolve, the treatment behavior of orthopedic science is gradually developing toward personalization and precision, among which 3D printing technology is an effective means to achieve this transformation (30). 3D printing technology has been successfully applied to many orthopedic fields, such as spinal screw placement, joint replacement, osteotomy, and orthopedics, all of which have achieved good results and application value (31–35). Through the 3D-printed personalized surgical guide, the OBS group achieves precise placement of screws and then combines the advantages of OBS to obtain the best treatment results. With the popularization of 3D printing technology, orthopedics’ treatment level and surgical quality will be significantly improved.

In summary, the lateral wall is crucial in treating intertrochanteric fractures, especially AO/OTA type 31-A3 femoral intertrochanteric fractures in elderly patients. OBS is recommended as the treatment of choice. Although this study found that PFNA has the benefits of shorter operative time and less bleeding, OBS leads to better postoperative recovery. Furthermore, a combination of OBS and PFNA was used in some cases to integrate their respective advantages and achieve satisfactory outcomes. However, the current number of cases remains limited. We are actively accumulating additional cases and anticipate that this combination will offer novel insights for clinical treatment moving forward. In clinical practice, proficiency in multiple internal fixation surgical techniques is crucial. One should select the most appropriate device and procedure by assessing the patient’s circumstances. Additionally, meticulous attention should be given to perioperative treatment and care to deliver optimal medical services (36).

## Data availability statement

The original contributions presented in this study are included in this article/supplementary material, further inquiries can be directed to the corresponding authors.

## Ethics statement

The studies involving human participants were reviewed and approved by the Yan’an Hospital affiliated to Kunming Medical University Ethical Committee (2022-182-01). Written informed consent for participation was not required for this study in accordance with the national legislation and the institutional requirements.

## Author contributions

ZC, HY, and JH designed the study. YX, JLi, CG, and JLu conducted the study and including the data collection. YR, ZY, XG, AZ, and JH analyzed the data. JH and MM wrote the manuscript. MM and AZ revised the manuscript. All authors contributed to the final approval of manuscript.
